# Antimicrobial Resistance, Virulence, and Genomic Characteristics of *Klebsiella pneumoniae* Isolated From Four Host Sources in Northeast China

**DOI:** 10.1155/tbed/1252580

**Published:** 2026-04-08

**Authors:** Qiu Xu, Cuilong Fan, Yaowei Liu, Xiaofeng Lu, Wei Song, Susu Du, Yao Zhu, Fang Xie, Wanjiang Zhang

**Affiliations:** ^1^ State Key Laboratory of Animal Disease Control and Prevention, Harbin Veterinary Research Institute, Chinese Academy of Agricultural Sciences, Harbin, 150069, China, caas.cn

**Keywords:** antimicrobial resistance, genotyping, *Klebsiella pneumoniae*, virulence, whole-genome sequencing

## Abstract

*Klebsiella pneumoniae* is an important emerging pathogen leading to serious clinical outcomes as the multidrug‐resistant (MDR) isolates arise. While *K. pneumoniae* is a well‐known pathogen in human clinical settings, its risks in nonhuman hosts and potential for cross‐species transmission remain poorly understood. In this study, we performed whole‐genome sequencing on 112 *K. pneumoniae* isolates from four host sources in Northeast China to elucidate their phylogenetic relationships and characterize their antimicrobial resistance (AMR) and virulence profiles. Our analysis identified 53 distinct sequence types (STs), 38 K‐locus (KL) types, and nine O‐loci. Notably, several strains with identical ST–KL combinations were shared across different hosts: ST76–KL5 was detected in both human and dairy cow sources; ST412–KL57 was found in human and chicken; ST111–KL63 was present in both pig and dairy cow sources, suggesting potential interspecies transmission. Antimicrobial susceptibility testing (AST) showed that nearly all human‐ and pig‐derived isolates were resistant to β‐lactams, quinolones, co‐trimoxazole, tetracyclines, and at least one aminoglycoside. In contrast, isolates from chickens and dairy cows exhibited lower resistance rates to these antibiotics. Overall, 88 of the 112 isolates (78.6%) were classified as MDR phenotype. Virulence gene screening indicated that 34 isolates carried the *iucABCD*/*iutA* aerobactin synthesis cluster, a key marker for hypervirulence. These *iuc*‐positive strains fell into two distinct phylogenetic lineages, including *iuc1* and *iuc3*. It is necessary to conduct the regular monitoring of the antibiotic resistance and hypervirulence of clinical *K. pneumoniae* from animals to prevent and control the transmission of *K. pneumoniae* along the food chain.

## 1. Introduction

*Klebsiella pneumoniae* is an important gram‐negative bacterium belonging to the Enterobacteriaceae family. While it is a normal part of the human gut microbiota, *K. pneumoniae* has evolved into diverse strains with varying antimicrobial resistance (AMR) and virulence profiles. It is classified as a critical priority healthcare‐associated pathogen by the World Health Organization (WHO) and serves as a major driver of AMR worldwide. As a bacterial pathogen thriving across multiple hosts, *K. pneumoniae* inhabits not only humans but also animals (mainly chicken, pig, and dairy cows) and wide range of environments (surface water, plants, soil, sewage, as well as domestic and wild animals) [1]. In livestock and poultry, *K. pneumoniae* is a significant pathogen responsible for pneumonia, bloodstream infections, and mastitis. Furthermore, it can cause mastitis in dairy cows and respiratory disease in pigs and chickens with high mortality rates [2]. In recent years, reported isolation rates from animals ranged from 9% to 35% [3–5].

*Klebsiella pneumoniae* is among the bacterial species that have shown a significant increase in resistance to most antibiotics [6]. Especially with the wide application of β‐lactam antibiotics, *K. pneumoniae* isolates capable of producing extended‐spectrum β‐lactamase (ESBL) are also increasing [5]. Additionally, resistance to aminoglycosides and fluoroquinolones has also been widely reported [7]. Infectious disease caused by carbapenem‐resistant *K. pneumoniae* (CRKP) has posed a serious public health threat [8, 9]. These strains exhibit multidrug‐resistant (MDR) phenotype, making infections increasingly challenging to treat. Notably, even without the use of carbapenems in livestock, CRKP is increasingly found in animal populations, raising concerns about its transmission between animals and humans [10]. Although antibiotic resistance was once largely attributed to clinical misuse, the use of antibiotics in agriculture and veterinary settings is now recognized as a major driver. In intensive farming systems, antibiotics are commonly used for therapy, disease prevention, and growth promotion [11]. These practices are strongly linked to high levels of AMR in humans [12]. Animals are considered important reservoirs of resistance genes, and resistant bacteria can spread to humans through the food chain or direct contact [13].

In addition to AMR, the emergence of hypervirulent *K. pneumoniae* (HvKp) is also a growing concern. HvKp efficiently acquires iron and overproduces capsular polysaccharide, resulting in a hypermucoid phenotype that is identified by a positive string test and is associated with severe disease and high mortality [14]. This phenotype is often attributable to a large virulence plasmid carrying capsular regulatory genes (*rmpA* and *rmpA2*) and multiple iron‐acquisition systems [15, 16]. Since its discovery in a patient with a liver abscess in Taiwan in the 1980s [14], HvKP has been increasingly isolated from both humans and animals, posing a considerable public health risk.

*Klebsiella pneumoniae* is not only transmitted among humans but also is commonly detected in animals [3, 17]. This widespread presence promotes the co‐dissemination of AMR and virulence genes across different hosts. Recent studies have shown that identical sequence types (STs) and mobile elements are present in both human and animal isolates, highlighting two‐way transmission between animal and human isolates [18]. However, most existing studies primarily focus on clinical *K. pneumoniae* isolates from humans; the genetic relationships between *K. pneumoniae* strains of animal and human origin remain poorly understood. We collected 112 *K. pneumoniae* isolates from different hosts and performed whole‐genome sequencing to analyze their phylogenetic relationships, virulence genes, AMR genes (ARGs), and plasmid replicon types. This study provides a foundation for assessing the risks of transmission between animals and humans.

## 2. Materials and Methods

### 2.1. Bacterial Strains

A total of 112 *K. pneumoniae* isolates were collected from hospitals, dairy farms, pig farms, and poultry farms in Northeast China. All isolates were stored at −80°C. Each sample was streaked onto MacConkey (Hopebio, China) agar plates and incubated at 37°C for 24 h. Suspect colonies of *K. pneumoniae* showing pale pink coloration were selected and inoculated on Luria–Bertani (LB; Coolaber, China) at 37°C for further examination. Identification and confirmation of the isolates were carried out using PCR for the *khe* gene [19] and *16S rRNA* gene sequencing.

### 2.2. Antimicrobial Susceptibility Testing (AST)

All *K. pneumoniae* isolates were tested for antimicrobial susceptibility by the Kirby–Bauer disk diffusion method, following Clinical and Laboratory Standards Institute (CLSI) recommendations (CLSI, 2020: M100 S30). For polymyxin and tigecycline, AST was performed using the broth dilution method, with results interpreted according to European Committee on Antimicrobial Susceptibility Testing (EUCAST) standards (available at: http://www.eucast.org/clinical_breakpoints/). *Escherichia coli* ATCC 25922 was used as the quality control strain. The tested antimicrobial agents included β‐lactams (amoxicillin, cefepime, and aztreonam), carbapenems (meropenem), aminoglycosides (gentamicin and amikacin), tetracyclines (doxycycline and tigecycline), fluoroquinolones (ciprofloxacin), sulfonamides (trimethoprim/sulfamethoxazole), and polymyxins (polymyxin B). The MDR phenotype is defined as nonsusceptibility to at least one agent in three or more antimicrobial categories [20].

### 2.3. Whole‐Genome Sequencing and Bioinformatic Analysis

The genomic DNAs of the *K. pneumoniae* isolates were purified using the Bacterial Genomic DNA Extraction Kit (TIANGEN, China), following the protocol provided by the manufacturer. The concentration and quantity of genomic DNA were measured using the NanoDrop 2000 spectrophotometer (Thermo Fisher Scientific, U.S.) and gel electrophoresis. Sequencing was performed on the Illumina NovaSeq platform (Illumina, USA). After sequencing, raw read quality was evaluated with FastQC v0.11.8 [21]. Trimmomatic v0.39 was used to remove Illumina adapters, low‐quality bases, and reads shorter than 36 bp under default parameters [22]. FLASh v1.2.11 was applied to merge overlapping paired‐end reads from short DNA fragments (shorter than twice the read length) into longer single‐end reads [23]. High‐quality reads were assembled de novo using SPAdes v3.9 [24]. Multilocus sequence typing was conducted with Kleborate (https://github.com/katholt/Kleborate), based on the seven housekeeping genes (*gapA*, *infB*, *mdh*, *pgi*, *phoE*, *rpoB*, and *tonB*). Capsular polysaccharide types (K‐locus [KL] types) and lipopolysaccharide (O) locus types were identified using Kaptive (https://github.com/katholt/kaptive). A minimum spanning tree of STs was generated with PHYLOVIZ [25]. ARG and virulence genes were predicted using ResFinder and the Virulence Factor Database (VFDB) [26, 27], respectively, with a sequence similarity threshold of >90%. The presence of plasmid replicons was identified using the PlasmidFinder database [26]. An SNP phylogenetic tree was constructed with Parsnp software and visualized using the iTOL online tool [28].

### 2.4. Hypermucoviscosity Testing

Hypermucoviscosity was measured by string test, as previously described [29]. In brief, *K. pneumoniae* isolates were grown overnight at 37°C on LB agar. A colony was lifted with a loop to evaluate the formation of a viscous string between the loop and the colony. A positive string test was defined as a string length of at least 5 mm.

## 3. Results

### 3.1. Prevalence and Phylogenetic Relationships of *Klebsiella pneumoniae* Isolates From Diverse Sources

A total of 112 *K. pneumoniae* strains were isolated from four host sources, including 47 from humans, 35 from pigs, 20 from dairy cows, and 10 from chickens (Figure 1A). Whole‐genome sequencing was performed on all 112 isolates, with all genomes successfully assembled. To elucidate the phylogenetic relationships among these *K. pneumoniae* isolates, we constructed a phylogenetic tree comprising 112 isolates. The analysis revealed that the isolates segregated into three major clades. Extensive intermingling of strains from different sources was observed throughout the phylogenetic tree, with no apparent segregation into distinct lineages based on their origin. For instance, the pig‐derived strain KPP34 clustered with the dairy cow‐derived KPB15, and the human‐derived strain KPH2 grouped with the dairy cow‐derived KPB1. Additionally, a cluster containing chicken‐derived strains (e.g., KPC1, KPC4, and KPC10) and human‐derived strains (e.g., KPH41, KPH25, and KPH7) was observed (Figure 1B). These findings suggest that the close phylogenetic relationship between isolates from different hosts may be a consequence of recent cross‐species transmission events.

Figure 1Host distributions and phylogenetic tree of the 112 strains of *Klebsiella pneumoniae*. (A) Host distributions of the samples. (B) Phylogenetic relationship of the dataset of 112 host‐derived *K. pneumoniae* genomes based on core‐genome SNPs with Parsnp. ST, KL type, O‐locus, and source are indicated by colored squares.

**Figure:**
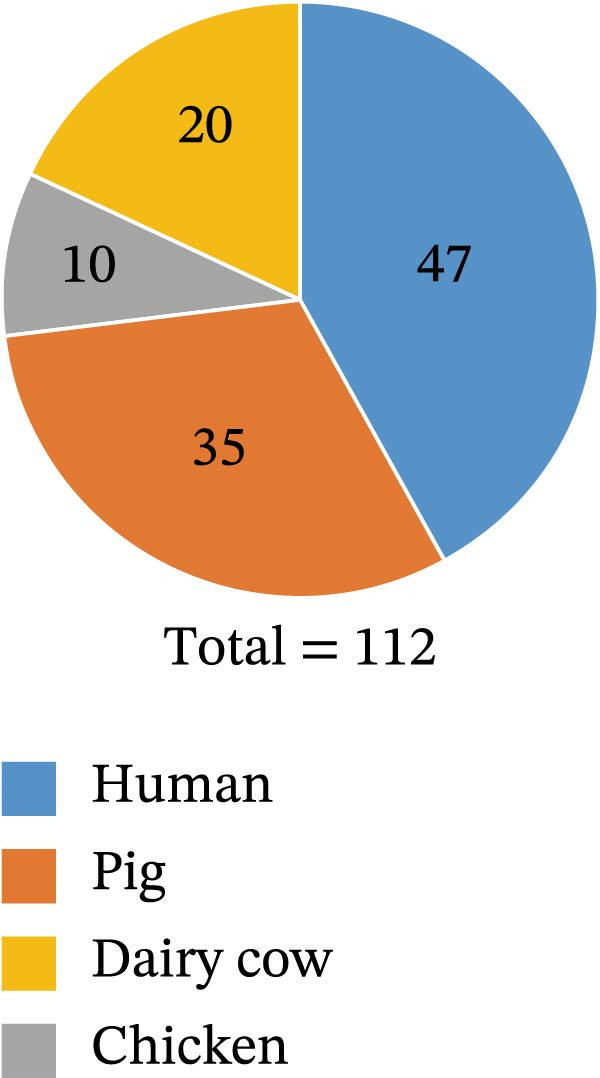
(A)

**Figure:**
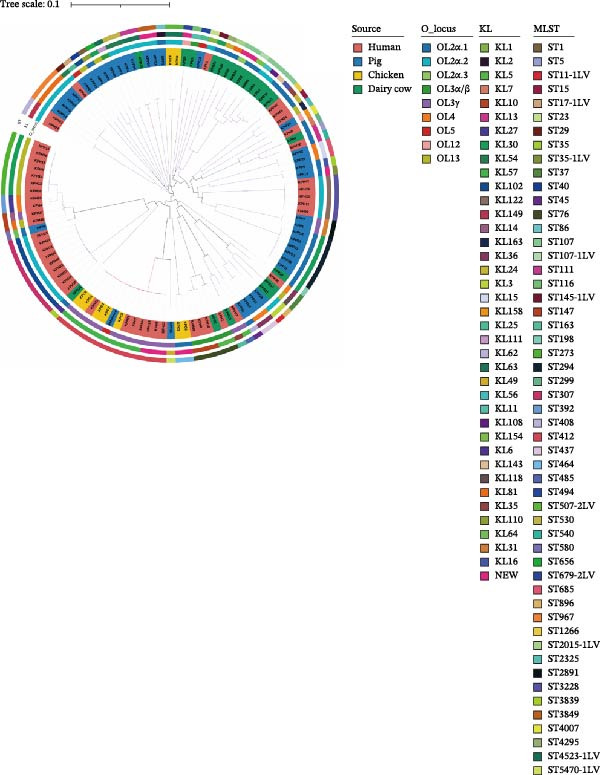
(B)

### 3.2. Molecular Typing of *Klebsiella pneumoniae* Isolates

**Figure 2:**
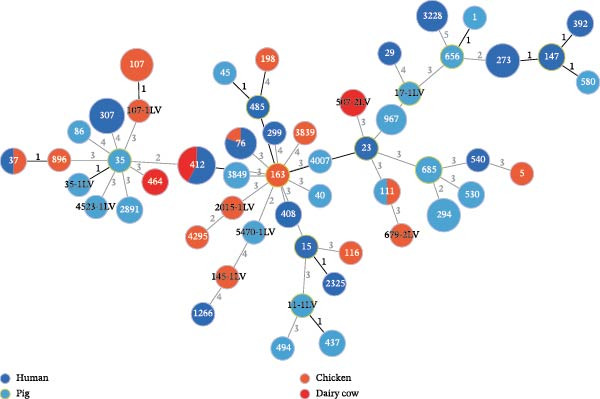
Molecular characteristics of the 112 strains of *Klebsiella pneumoniae*. PHYLOVIZ analysis showing the STs’ relationship based on the GoeBURST algorithm among 112 *K. pneumoniae* isolates. Colors represent sources, and node sizes are proportional to the number of isolates. The numbers on the lines represent the number of allele differences between the two connected nodes.

A total of 53 STs were identified among the 112 *K. pneumoniae* isolates. ST412 was the most prevalent (12/112, 10.7%), followed by ST307 (9/112, 8.0%), ST273 (7/112, 6.3%), ST107 (6/112, 5.4%), ST294 (6/112, 5.4%), ST76 (5/112, 4.5%), ST3228 (5/112, 4.5%), and ST967 (4/112, 3.6%). Phylogenetic analysis of MLST profiles was performed based on the geoBURST algorithm. Distinct STs were predominant across different hosts. ST307 was predominant in human‐derived strains (9/47). Among animal‐derived strains, the predominant STs were ST294 in pigs (6/35), ST107 in dairy cows (6/20), and ST412 in chickens (4/10). In addition, evidence of potential cross‐species transmission was observed, with several STs shared between hosts: ST76 and ST37 were shared between humans and dairy cows; ST412 was common to humans and chickens; ST111 was identified in both pigs and dairy cows (Figure 2). A total of 38 distinct KL types were identified by capsular typing among the 112 isolates, 10 of which represented novel types. Among these, the most prevalent capsular types were KL30 (16 isolates), KL102 (10 isolates), KL57 (nine isolates), and KL81 (six isolates). KL1 and KL2 are typically associated with hypervirulent strains; one human‐derived KL1 and one pig‐derived KL2 were identified in this study. Through ST, KL, and source‐relatedness analyses, this study detected ST76 and KL5 phylotypes in both human and dairy cow sources, ST412 and KL57 in both human and chicken sources, and ST111 and KL63 in both pig and dairy cow sources (Figure 1B). In addition, nine distinct O‐ loci were identified, including OL2α.1 (28 isolates), OL2α.2 (34 isolates), OL3γ (18 isolates), OL13 (12 isolates), OL4 (seven isolates), OL5 (two isolates), OL12 (two isolates), and OL2α.3 (one isolate). Among them, OL2α.2 was the predominant type. Importantly, OL2α.2, OL2α.1, and OL3γ were present in all four host species examined, and OL5 was exclusively found in pig‐derived strains (Figure 1B). These findings indicate that the shared strains pose a risk of cross‐species transmission, suggesting that horizontal transmission between humans and animals is a potential route.

### 3.3. Antimicrobial Susceptibility Profiles and Detection of Resistance Genes

The AMR profiles of *K. pneumoniae* isolates were investigated. AST results demonstrated that isolates from human (91%) and pig (85%) exhibited resistance to β‐lactams, quinolones, co‐trimoxazole, tetracyclines, and aminoglycosides. In contrast, isolates from chickens and dairy cows, while resistant to β‐lactams and quinolones, remained largely susceptible to tetracyclines and aminoglycosides. Notably, all chicken‐ and dairy cow‐derived isolates were susceptible to co‐trimoxazole (Figure 3A). Among the 112 *K. pneumoniae* isolates, 88 (78.6%) were identified as MDR strains. The prevalence of MDR was highest among pig‐derived strains (97.1%), followed by human‐derived strains (95.7%), chicken‐derived strains (60%), and dairy cow‐derived strains (20%; Figure 3B).

Figure 3Antimicrobial resistance phenotypes of the 112 strains of *Klebsiella pneumoniae* from various host species. (A) Heatmap of the antimicrobial susceptibility profile of 112 isolates of *K. pneumoniae*. Resistance (blue) or susceptibility (red) against a particular antibiotic is defined using the EUCAST clinical breakpoint at the time of isolation. AK, amikacin; AMC, amoxicillin; ATM, aztreonam; CIP, ciprofloxacin; CRO, ceftriaxone; CT, colistin; DO, doxycycline; GEN, gentamycin; MEM, meropenem; SXT, trimethoprim/sulfamethoxazole; TGC, tigecycline. (B) Proportion of isolates from different hosts that are resistant to 1–7 classes of antibiotics.

**Figure:**
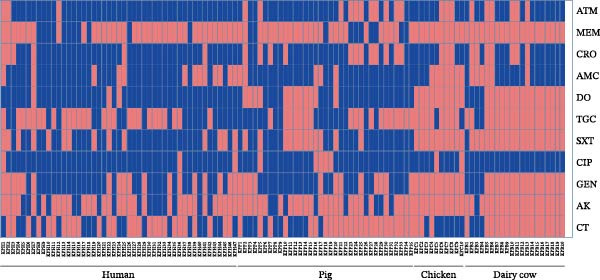
(A)

**Figure:**
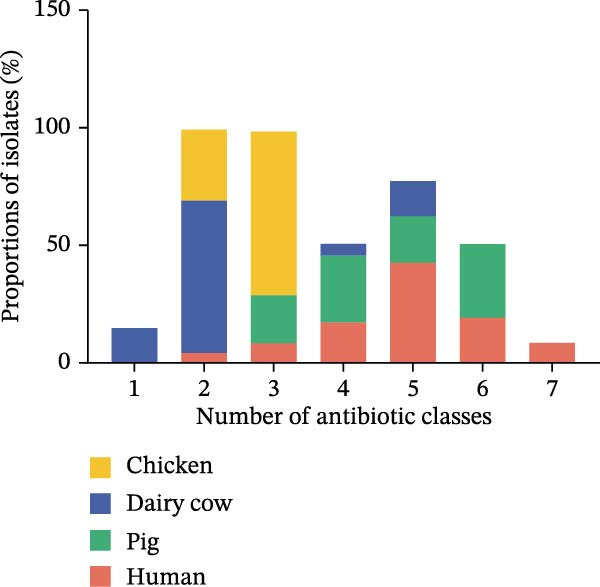
(B)

A total of 45 ARGs (including all *bla*_SHV_ subtypes) were identified by ResFinder. The dominant resistance genes were *oqxAB*, *fosA*, *aph*(*3^′^*)*-Ib-aph*(*6*)*-Id*, *sul1*, *dfrA*, *tet*(A), *mph*(A), *bla*_SHV_, and *floR*. Notably, the *oqxAB* and *fosA* genes were present in all 112 isolates (Figure 4A). ARGs for β‐lactams, fosfomycin, and quinolones were detected across all four hosts (Figure 4B). Resistance gene distribution exhibited host‐specific patterns. Isolates from humans and pigs harbored a significantly greater number and diversity of resistance genes compared to those from chickens and dairy cows (Figure 4C). Among chicken‐derived isolates, only three resistance genes, *bla*_SHV_, *fosA*, and *oqxAB*, were detected (Figure 4A). Notably, carbapenem resistance genes were identified in isolates from both pigs and humans, with distinct profiles: *bla*_NDM−5/1_ was found in 10 pig‐derived isolates, *bla*_KPC−2_ in 11 human‐derived isolates, and *bla*_IMP−8_ in one human‐derived isolate, respectively. Among the carbapenem‐resistant isolates, the predominant STs are ST273 (14.89%, 7/47) of human origin and ST294 (17.14%, 6/35) of pig origin, with KL30 serving as the primary capsular type (Figure 4D).

Figure 4Phylogenetic tree and ARGs of the 112 strains of *Klebsiella pneumoniae*. (A) Construction of a core genome SNPs phylogenetic tree based on 112 *K. pneumoniae* strains. The presence of AGRs is indicated by different colors. (B) A heatmap showing the percentage of different sources carrying at least one antimicrobial resistance gene for each class of antibiotics. (C) Number of antimicrobial resistance genes from different sources. (D) Proportional distribution of distinct STs and KL types among carbapenem‐resistant strains.

**Figure:**
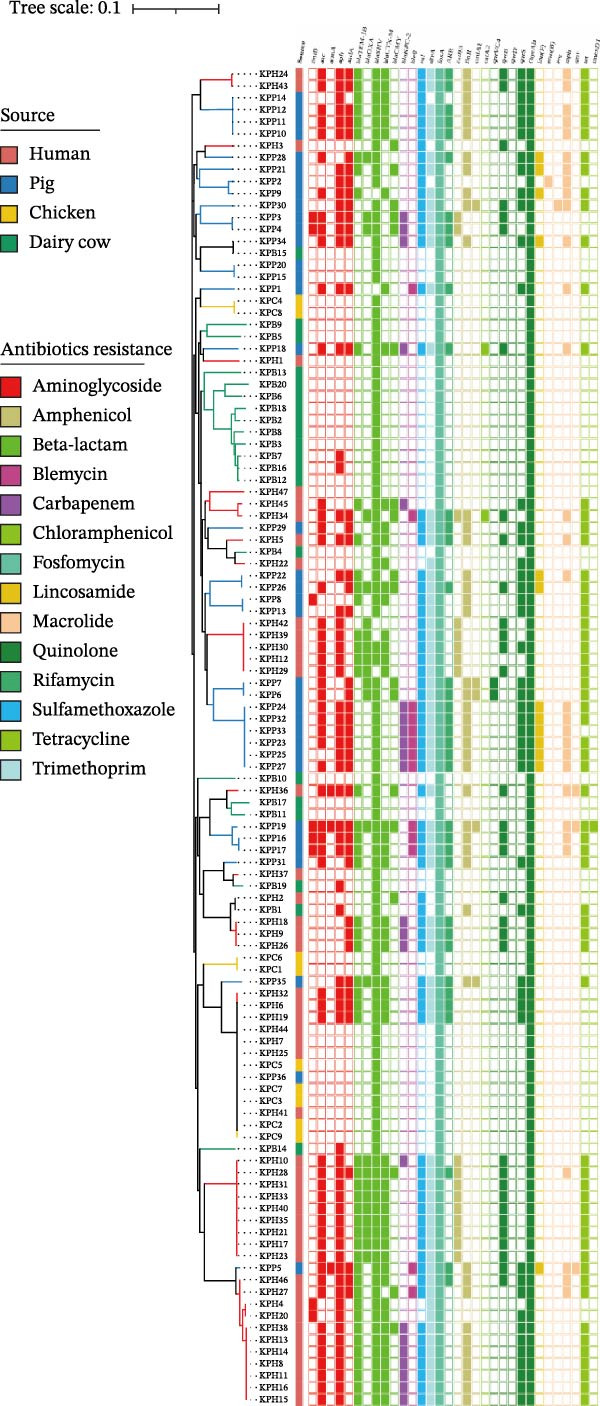
(A)

**Figure:**
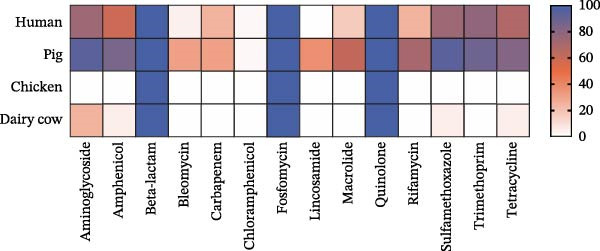
(B)

**Figure:**
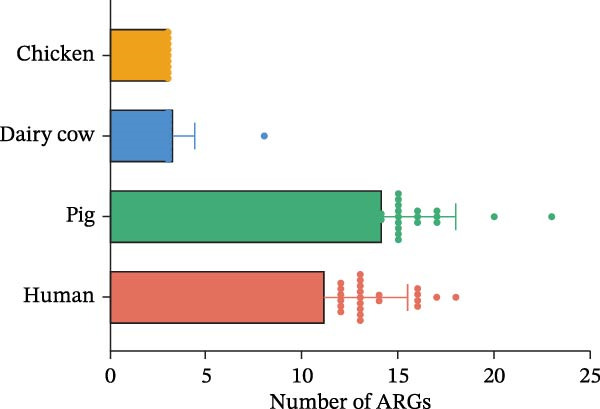
(C)

**Figure:**
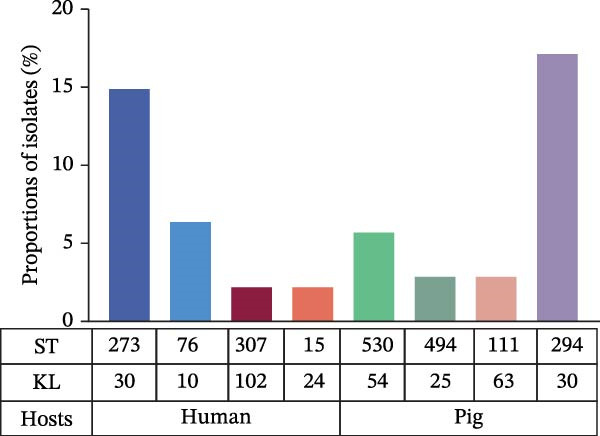
(D)

### 3.4. Virulence Gene Profiles of *Klebsiella pneumoniae* Isolates

The hypervirulent phenotype was predominantly associated with two contributing factors: mucoid regulators and siderophores. Hypervirulence‐associated hypermucoviscosity was primarily mediated by the mucoid regulator genes, namely *rmpA* and *rmpA2*. Siderophores are iron‐chelating molecules secreted by bacteria to facilitate iron acquisition from the environment [14, 30, 31]. We identified 36 isolates carrying one or more of hypervirulence genes (*iucA*, *iroB*, *rmpA*, *rmpA2*, *cbl*, and *ybt*). In this study, 17 strains simultaneously contained four hypervirulence genes (*iucA*, *iroB*, *rmpA*, and *rmpA2*), distributed across human (*n* = 10), chicken (*n* = 6), and pig (*n* = 1) hosts. These strains encompassed four STs (ST23, ST86, ST412, and ST29), as well as four capsular types (KL1, KL2, KL5, and KL57; Figure 5A). The gene *iuc* was identified as the most common iron carrier secreted by HvKp [32]. Among *K. pneumoniae* isolates, 30% (34/112) carried the *iuc* gene, which was exclusively classified into two lineages: *iuc1* and *iuc3*, each found in 17 isolates. No *iuc2* or *iuc5* variants were detected. The highest frequency of the *iuc* gene was detected in pig sources (Figure 5B). Kleborate‐assessed virulence scores and distribution of virulence genes revealed higher virulence in human isolates (Figure 5C,D). Only six string test‐positive strains were identified among the HvKp isolates (Figure 5A). This indicates that the presence of the *rmpADC* and *rmpA2* genes does not necessarily confer a hypermucoviscous phenotype (hmv).

Figure 5Virulence genes of the 112 strains of *Klebsiella pneumoniae*. (A) Phylogenetic tree of the dataset of 112 host‐derived *K. pneumoniae* genomes based on core‐genome SNPs with Parsnp. The presence of source and virulence genes is indicated. aerobactin biosynthesis genes: *iuc*; salmochelin biosynthesis genes: *iro*; yersiniabactin biosynthesis loci: *ybt*; colibactin biosynthesis loci: *clb*; mucoid regulator operon: *rmpADC*; mucoid regulator 2 genes: *rmpA2*; hypermucoviscosity: *hmv*. (B) Proportions of isolates harboring distinct aerobactin (*iuc*) lineages across different host sources. (C) Proportions of isolates carrying virulence genes across different host sources. (D) Proportions of isolates by Kleborate‐predicted virulence scores across different host sources.

**Figure:**
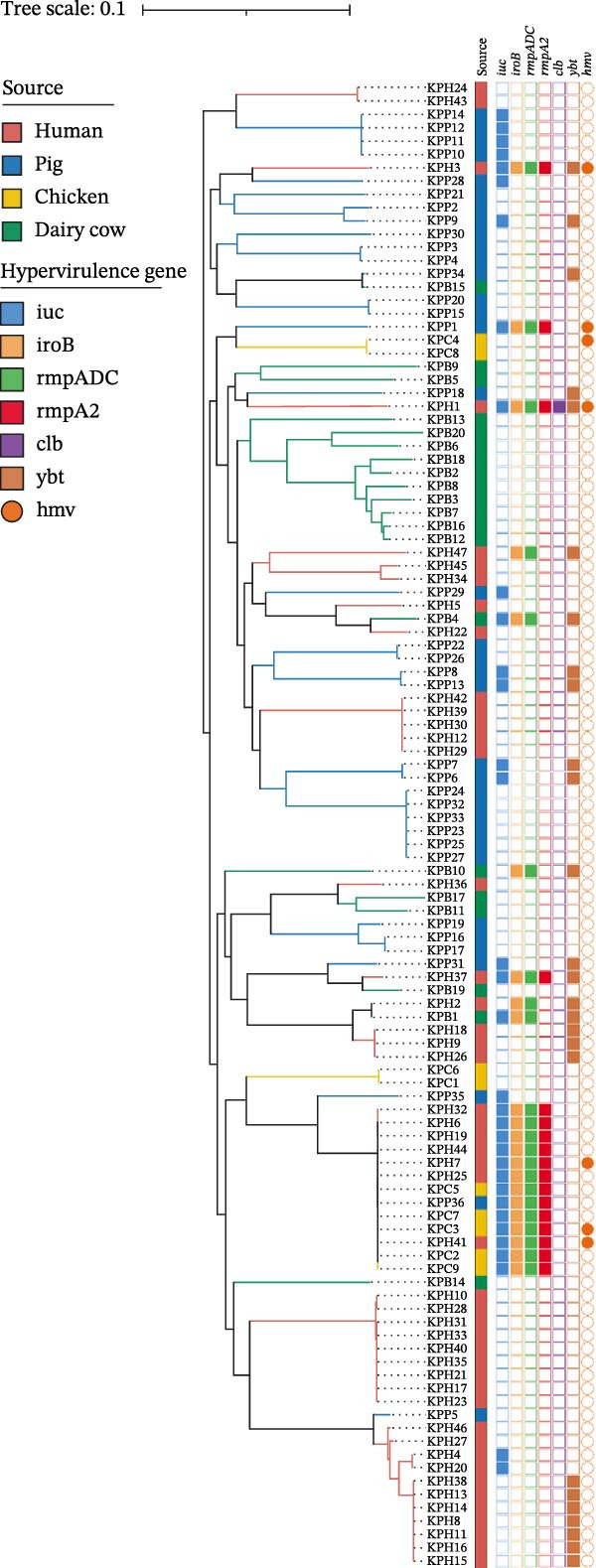
(A)

**Figure:**
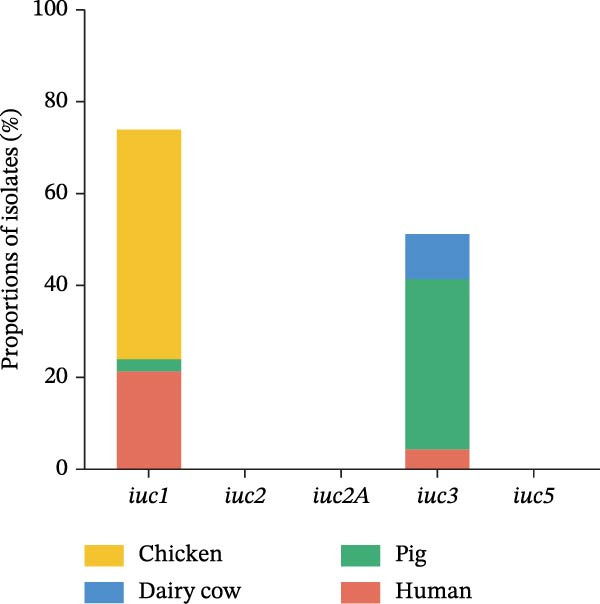
(B)

**Figure:**
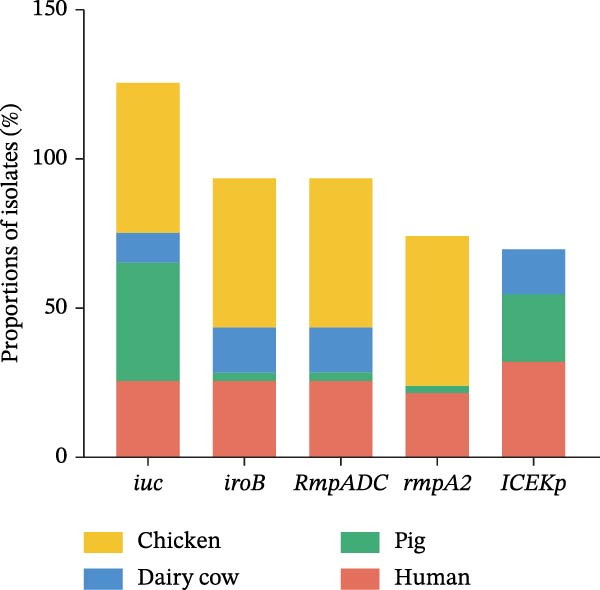
(C)

**Figure:**
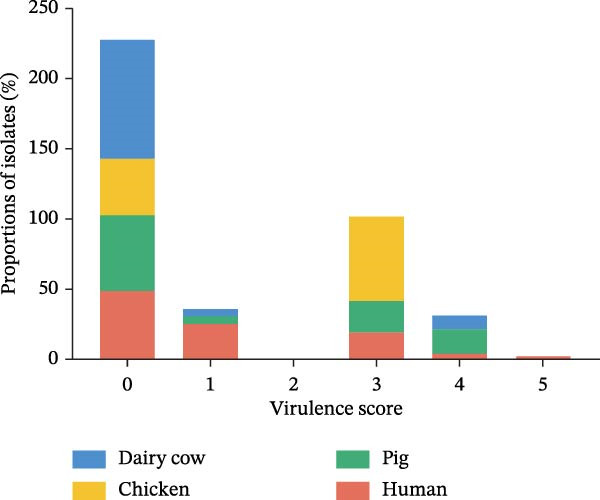
(D)

In this study, seven unique virulence gene profiles were detected, including *iuc*, *iuc/ybt*, *iroB/RmpADC/ybt*, *iuc/iroB/RmpADC/ybt*, *iuc/iroB/RmpADC/rmpA2*, *iuc/iroB/RmpADC/rmpA2/ybt*, and *iuc/iroB/RmpADC/rmpA2/ybt/cbl*. It is noteworthy that an inverse correlation was observed between the number of virulence genes and the number of AGRs (Figure 6A). Isolates harboring hypervirulence genes were derived from humans (*n* = 13), pigs (*n* = 14), chickens (*n* = 6), and dairy cows (*n* = 3). The ST412 clonal type was predominant among isolates (Figure 6B). The KL57 was predominant among isolates (Figure 6C).

Figure 6ARGs, ST, and KL of 36 *Klebsiella pneumoniae* isolates harboring genes encoding hypervirulence. (A) An UpSet plot showing the total number of ARGs, filled dots representing the presence of virulence genes, and different colors representing the number of resistance genes. (B) Proportions of isolates with distinct STs carrying hypervirulence genes across different host sources. (C) Proportions of isolates with distinct KL‐carrying hypervirulence genes among different host sources.

**Figure:**
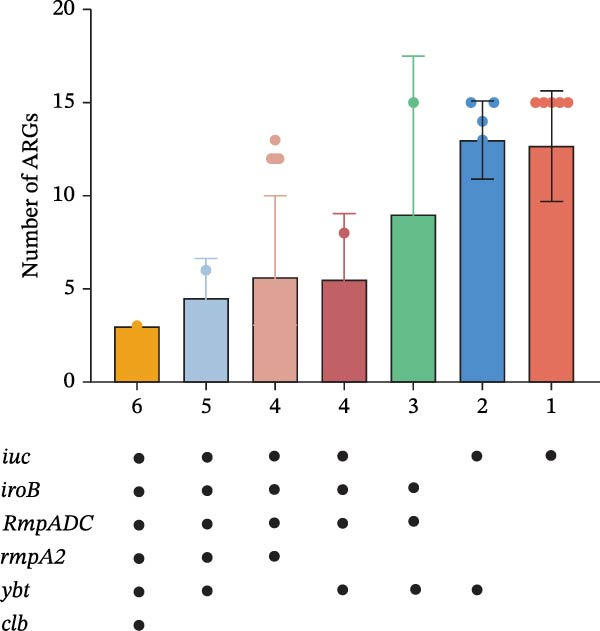
(A)

**Figure:**
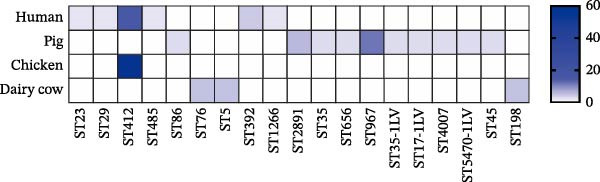
(B)

**Figure:**
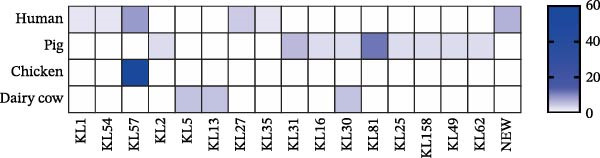
(C)

### 3.5. Characterization of Plasmid Replicon of *Klebsiella pneumoniae* Isolates

**Figure 7:**
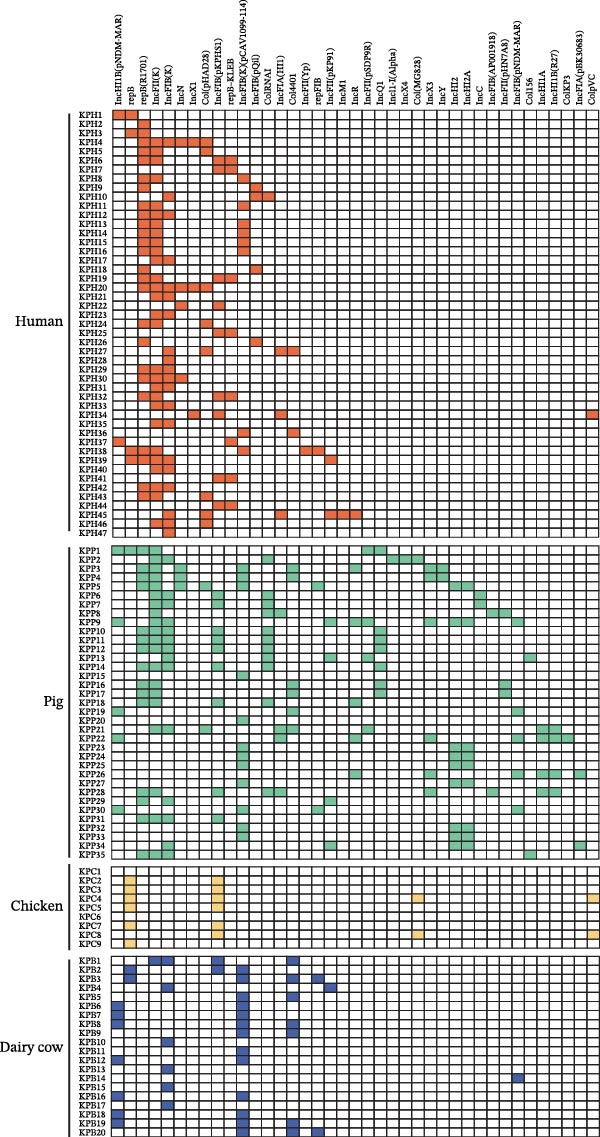
Distribution of plasmid replicons of *Klebsiella pneumoniae* isolates across different host sources. Heatmap of the plasmid replicon types of 112 isolates of *K. pneumoniae*. Orange, green, blue, and yellow indicate the presence of plasmid replicon types in different hosts, while white indicates the absence of plasmid replicon types.

The plasmid replicons in the *K. pneumoniae* isolates were analyzed using PlasmidFinder. A striking 98.2% (110/112) of the isolates harbored plasmid replicons. A total of 39 distinct plasmid replicon types were identified. Overall, plasmid replicons from animal‐derived isolates were more diverse than those from humans. Thirty‐five single replicon types were identified from animal isolates compared with 21 from human isolates. Among these, pig‐derived isolates exhibited the greatest diversity in replicon types, with 33 distinct replicon types identified. The predominant replicon type was IncFII(K) in both human (28/47, 59.5%) and pig (20/35, 57.1%) isolates. In contrast, IncFIB (pKPHS1) was most common in chicken isolates (7/10, 70.0%) and IncFIB(K) (pCAV1099‐114) was the predominant replicon type in dairy cow isolates (13/20, 65.0%). Fifteen replicon types were identified in isolates from both humans and animals. In addition, evidence of potential cross‐species transmission was observed, with several replicon types shared by distinct hosts. The replicon types repB and IncFIB (pKPHS1) were detected across four host species, and seven replicon types (IncHI1B (pNDM‐MAR), repB, IncFIB(K), IncFII(K), IncFIB(K)(pCAV1099‐114), Col440I, repFIB, and IncFII (pKP91)) were shared among isolates from humans, pigs, and dairy cows. Six replicon types (repB (R1701), IncN, Col (pHAD28), ColRNAI, IncFIA (HI1), and IncR) were shared between human and pig isolates. IncFIB (pNDM‐Mar) was shared between dairy cow and pig isolates. ColpVC was shared between human and chicken isolates (Figure 7).

## 4. Discussion

The emergence and transmission of MDR and HvKP strains can cause severe and difficult‐to‐treat infections in both humans and animals [33]. However, few studies have explored the phylogenetic relationships between animal and human sources [34]. This study investigated AGRs, virulence genes, and phylogenetic relationships in *K. pneumoniae* from different sources in Northeast China. This study isolated and identified 112 *K. pneumoniae* isolates, which included 53 distinct STs, 38 KL types, and nine O loci. These isolates exhibited extremely high genetic diversity, consistent with previous studies. For example, one study reported 61 STs and 51 KL types among 180 isolates in the United States [35], while another study in Hubei, China, detected 100 different STs among 239 isolates [36]. To date, there are currently 12 distinct O loci identified, with both the OL1 and OL2 antigens the most common [37, 38]. This study found that OL2α.2 was the predominant prevalent type, with the absence of OL1 locus. *K. pneumoniae* is widely distributed in livestock, companion animals, and the environment; these sources serve as potential reservoirs for human infections [39]. The close contact between animals and humans increases the risk of bacterial transmission across different species. Studies have demonstrated cross‐infection of *K. pneumoniae* strains between humans and companion animals, highlighting the potential for interspecies transmission [17, 40]. In addition, core genome phylogenetic analysis revealed that isolates from human and animal sources clustered on the same branches and shared identical STs and KL types, providing evidence for possible clonal transmission between humans and animals.

*Klebsiella pneumoniae* MDR strains pose a serious global public health threat. Its presence has been documented in numerous countries, including northwestern Iran, Turkey, Australia, and China [2, 33, 41, 42]. In this study, MDR strains were detected at a high frequency of 78.5%. Among these, isolates from both human and animal sources showed the highest resistance rates to ciprofloxacin. As broad‐spectrum antimicrobials, fluoroquinolones are widely used in livestock production in China, which may be one of the factors contributing to the high ciprofloxacin resistance rate [43, 44]. Notably, the ciprofloxacin resistance rate observed in chicken‐derived isolates in this study was 80.0%, which closely aligns with the previously reported rate of 81% for chicken‐derived *K. pneumoniae* in China [45]. Approximately 70% of antibiotics are used globally in agriculture, particularly in livestock farming [46]. We further analyzed the ARG burden across strains from different farm animals. Pig‐derived strains harbored the highest ARG burden, whereas chicken‐derived and dairy cow‐derived strains showed the lowest. This trend is consistent with the relatively lower antibiotic use in poultry and dairy cow farming compared to pig production [47].

Notably, this study identified 12 carbapenem‐resistant human isolates, mainly carrying *bla*_KPC−2_ and *bla*_IMP−8_. Although carbapenems are not approved for use in livestock, we detected 10 carbapenemase gene‐harboring strains (mostly *bla*_NDM_) from pigs. A previous study has also identified the presence of CRKP (primarily *bla*_NDM−5_) in swine farms [48]. Previous studies have also reported Enterobacteriaceae carrying carbapenem resistance genes (particularly *bla*_NDM_ and *bla*_OXA−48_) in poultry and pig farms, indicating a growing prevalence of carbapenemase genes among animal‐derived isolates [49]. The genetic relatedness of carbapenem resistance genes between human and animal reservoirs indicates the necessity of addressing AMR within the One Health framework.

The virulence genes, including *iroB*, *iucA*, *rmpA*, and *rmpA2*, are key biomarkers for HvKp. Typical HvKp strains belong to ST ST23 and serotypes KL1 or KL2 [50]. We identified an ST23 isolate of pig origin and one KL1 and one KL2 strain associated with high virulence. Consistent with previous reports, isolates carrying hypervirulence genes were predominantly ST412/KL57. For example, Wu et al. [36] described two ST412/KL57 HvKp strains causing gas gangrene and sepsis, noting that ST412 can acquire virulence and resistance genes via plasmid transfer. This study detected ST412/KL57 in both human and chicken samples, suggesting a possible risk of animal‐to‐human transmission. *Klebsiella pneumoniae* is recognized to induce severe infections in various animal species, such as piglet septicemia, bovine mastitis, and poultry respiratory infections, impacting animal health and agricultural economies [39]. We isolated seve animal‐derived strains carrying multiple hypervirulence genes, along with 24 others carrying the HvKp biomarker gene *iuc*. The *iuc* operon in *K. pneumoniae* plasmids comprises five known variants (*iuc1*, *iuc2*, *iuc2a*, *iuc3*, and *iuc5*), which exhibit distinct host‐specific distributions. Among these, *iuc1* is typically predominant in human isolates, whereas *iuc3* is more common in animal isolates [51]. Notably, the *iuc3* variant has been identified in human isolates, whereas *iuc1* has been detected in chickens and pigs. Wang et al. [52] reported *iuc3*‐positive HvKp in dairy cows, noting that *iuc3* plasmids showed high variability and transferability. Furthermore, we also found many animal‐derived strains carrying *iuc3* but lacking *rmpA*, highlighting the need for ongoing surveillance of *iuc3*‐positive plasmids. In this study, most isolates with the *rmpA* did not show a hypermucoviscous phenotype. This may be due to mutations in virulence genes or regulation by other genetic elements in the plasmid or chromosome.

Plasmids serve as the primary vehicle for disseminating virulence and ARGs among bacterial populations. Their duplication and survival capacity depend on their replication determinants (replicon) [53]. Notably, the IncFIB (pKPHS1)‐type plasmid was found across four host species. IncF plasmids play a significant role in disseminating ARGs and are often associated with the carriage of virulence genes in Enterobacteriaceae [54]. As a critical component of virulence plasmids, IncFIB‐type plasmids are closely linked to the formation and spread of MDR‐HvKp, thereby facilitating the convergence of resistance and hypervirulence [55]. Notably, in this study, IncR plasmids were found circulating in both human and pig hosts. It has been reported that IncR plasmids are characterized by carrying multiple ARGs, and their conserved backbones possess MDR regions capable of facilitating the acquisition of further resistance determinants [56].

## 5. Conclusions

In summary, our results provide information related to the genomic structure, molecular characteristics, virulence profile, ARGs, and plasmid replicons of *K. pneumoniae* isolates from different host sources. This study indicates that *K. pneumoniae* have the risk of horizontal transmission among humans and animals and are undeniably a one‐health problem. Therefore, it is imperative to implement the regular monitoring of the MDR of clinical *K. pneumoniae* from animals to prevent the transmission of *K. pneumoniae* along the food chain.

## Author Contributions

Qiu Xu, Yao Zhu, and Fang Xie contributed to writing of the manuscript. Cuilong Fan, Yaowei Liu, Xiaofeng Lu, Wei Song, and Susu Du contributed to the acquisition and analysis of the data. Yao Zhu, Fang Xie, and Wanjiang Zhang contributed to the study design and data interpretation.

## Funding

This work was supported by the Prevention and Control of Emerging and Major Infectious Diseases‐National Science and Technology Major Project (Grant 2025ZD01900100), the Heilongjiang Provincial Natural Science Foundation of China (Grant LH2024C061), the Central Public‐interest Scientific Institution Basal Research Fund (Grants 1610302024001 and 1610302024002), and the National Key Research and Development Program of China (Grant 2024YFC2607402).

## Ethics Statement

The authors have nothing to report.

## Conflicts of Interest

The authors declare no conflicts of interest.

## Data Availability

The data that support the findings of this study are openly available in the Pathogenwatch database (https://next.pathogen.watch/collections/61QUzVv8aD8tamEoKs5RHa-112-klebsiella-pneumoniae-strains).
